# Titanium Dioxide Nanoparticles Induced HeLa Cell Necrosis under UVA Radiation through the ROS-mPTP Pathway

**DOI:** 10.3390/nano10102029

**Published:** 2020-10-15

**Authors:** Runqing Geng, Yuanyuan Ren, Rong Rao, Xi Tan, Hong Zhou, Xiangliang Yang, Wei Liu, Qunwei Lu

**Affiliations:** 1 College of Life Science and Technology, Huazhong University of Science and Technology, Wuhan 430074, China; runqinggeng@mail.hust.edu.cn (R.G.); renyuanyuan@mail.hust.edu.cn (Y.R.); raorong_2004@126.com (R.R.); xtan@mail.hust.edu.cn (X.T.); hz199268@mail.hust.edu.cn (H.Z.); yangxl@mail.hust.edu.cn (X.Y.); 2 National Engineering Research Center for Nanomedicine, Huazhong University of Science and Technology, Wuhan 430074, China; 3 Key Laboratory of Molecular Biophysics of the Ministry of Education, College of Life Science and Technology, Center for Human Genome Research, Huazhong University of Science and Technology, Wuhan 430074, China

**Keywords:** nano-TiO_2_, UVA, cell necrosis, reactive oxygen species, mitochondrial permeability transition pore

## Abstract

Titanium dioxide nanoparticles (nano-TiO_2_), as a common nanomaterial, are widely used in water purification, paint, skincare and sunscreens. Its safety has always been a concern. Prior studies have shown that ultraviolet A (UVA) can exacerbate the toxicity of nano-TiO_2_, including inducing cell apoptosis, changing glycosylation levels, arresting cell cycle, inhibiting tumor cell and bacterial growth. However, whether the combination of UVA and nano-TiO_2_ cause cell necrosis and its mechanism are still rarely reported. In this study, we investigated the cytotoxicity and phototoxicity of mixture crystalline nano-TiO_2_ (25% rutile and 75% anatase, 21 nm) under UVA irradiation in HeLa cells. Our results showed that the abnormal membrane integrity and the ultrastructure of HeLa cells, together with the decreased viability induced by nano-TiO_2_ under UVA irradiation, were due to cell necrosis rather than caspase-dependent apoptosis. Furthermore, nano-TiO_2_ and UVA generated the reactive oxygen species (ROS) and caused the mitochondrial permeability transition pore (mPTP) of HeLa cells to abnormally open. Cell viability was significantly increased after adding vitamin C (VC) or cyclosporin A (CsA) individually to inhibit ROS and mPTP. Clearance of ROS could not only impede the opening of mPTP but also reduce the rate of cell necrosis. The results suggest the possible mechanism of HeLa cell necrosis caused by nano-TiO_2_ under UVA irradiation through the ROS-mPTP pathway.

## 1. Introduction

Titanium dioxide (TiO_2_) is a white insoluble powder because of its brightness and high refractive index and is used as an additive in numerous products to make them white [1]. Compared with fine particles, titanium dioxide nanoparticle (nano-TiO_2_) is widely used in food additives, sunscreen, water quality restoration, drug delivery and photosensitizer of photodynamic therapy due to its good biocompatibility, strong stability, photocatalytic property, and intense sensitivity to heat and magnetism [2,3,4]. However, the intended use of nano-TiO_2_ shows controversy regarding its unique properties, which have always been a concern.

Prior studies by our group have proven that nano-TiO_2_ can penetrate through the stratum corneum of pig ears into a deep layer of the epidermis [5]. Ultraviolet A (UVA) is the main component of ultraviolet (UV) in sunlight, up to 95%, which can penetrate the dermis [6]. Under ultraviolet irradiation, electrons in the valence band of nano-TiO_2_ absorb light energy and jump to the conduction band, leaving valence band holes, extracting electrons from water or hydroxyl ions, and producing reactive oxygen species (ROS) [7,8]. ROS produced by nano-TiO_2_ under UV irradiation destroyed cell or tissue homeostasis by oxidizing lipid, protein, nucleic acid, or other biomolecules, leading to genotoxicity, cytotoxicity, and pathological change [9,10]. It was recently demonstrated that nano-TiO_2_ under UVA irradiation-induced keratinocytes (HaCaT cells) apoptosis via ROS generation, thereby opening the mitochondrial permeability transition pore (mPTP), decreasing mitochondrial membrane potential, activating caspase-3, and initiating cell death signal [11]. However, it has been reported that oxidative stress and mitochondrial damage trigger cell necrosis in HeLa cells [12].

Apoptosis and necrosis are two kinds of cell death modes. Apoptosis could be identified by morphological observation and landmark proteins. Typical apoptotic bodies in A549 cells treated with nano-TiO_2_ were observed by scanning electron microscope [13]. Administration of nano-TiO_2_ for 30 days, the gene expressions of bax and p53 were elevated, bcl-2 expression was reduced, and cleaved caspase-3 activity was increased in the intestinal and liver of rats, which suggested that nano-TiO_2_ induced apoptosis [14]. Cell necrosis is contrasted with apoptosis, characterized by cell swelling and membrane rupture [15]. ZnO nanoparticles caused the acute cytoskeletal collapse and damaged cell membrane integrity, triggering necrosis [16]. Necrosis could be induced by various stimuli, such as trauma, exposure to toxic substances, local ischemia, virus or bacterial infection [17]. Rapid growing evidence has shown that nano-TiO_2_ exposure can also lead to cell necrosis and dysfunction. Wang et al. reported that nano-TiO_2_ with different particle sizes caused spotted necrosis of hepatocytes around the central vein in female mice [18]. It has been demonstrated nano-TiO_2_ can accumulate in the kidney, leading to cell necrosis and dysfunction [1]. Higher doses of nano-TiO_2_ can cause Sertoli cells autophagy and necrosis, and damage spermatogenic cells and testis of zebrafish [19]. Despite many studies on the phenomenon of necrosis induced by nano-TiO_2_, the underlying mechanism remains unclear. Therefore, it is necessary to analyze the relationship between the phototoxicity of nano-TiO_2_ and cell necrosis and the molecular mechanism for its safe application.

Oxidative stress is one of the main mechanisms involved in nanoparticle-induced necrosis by reducing mitochondrial membrane potential and damaging the mitochondria. mPTP is a non-specific channel between the inner and outer membrane of mitochondria, which plays a vital role in regulating mitochondrial membrane potential [20]. Some studies have proposed the critical function of this mitochondrial channel complex in regulating cell necrosis. P53 transferred to mitochondria and triggered the opening of mPTP through interaction with Cyclophilin D (CypD) under the stimulation of oxidative stress, which led to cell necrosis [21]. Cells or mice lacking CypD can avoid necrosis under hypoxia, calcium overload and oxidative stress [22]. These results indicate that mPTP plays a vital role in the process of cell necrosis. Taking this into consideration, we speculate that a large amount of ROS produced by UVA and nano-TiO_2_ may induce necrosis, but the specific mechanism needs to be further explored.

This study aims to investigate the cytotoxicity of nano-TiO_2_ and UVA on HeLa cells and its mechanism. It demonstrated the decrease of HeLa cell viability induced by nano-TiO_2_ under UVA irradiation, not caspase-dependent apoptosis but necrosis for the first time. Furthermore, the results revealed the possible mechanism of cell necrosis regulated by ROS-mPTP pathway with the co-treatment of nano-TiO_2_ and UVA irradiation. Understanding toxic mechanisms is essential for the safe application of nanoparticles. On the other hand, it could be exploited in the treatment of diseases.

## 2. Materials and Methods

### 2.1. Chemicals and Materials

Nano-TiO_2_ P25 was purchased from Degussa Company (Essen, Germany). The physical and chemical characteristics are as follows: purity ≥ 99%; crystal structure of 25% rutile and 75% anatase; particle diameter is about 21 nm; particle specific surface area is about 50 m^2^/g). UVA was provided by UV lamp (ZF-5, 365 nm, 8 W, 0.6 mW/cm^2^, Shanghai Huxi Instrument, Shanghai, China).

Tetramethylrhodamine ethyl ester (TMRE), Dulbecco’s modified Eagle medium (DMEM), Fetal bovine serum (FBS), phosphate-buffered saline (PBS, pH 7.4), penicillin, streptomycin, and trypsin-EDTA were purchased from Gibco (Invitrogen, Carlsbad, CA, USA). Thiazolyl blue (MTT), 2′,7′-dichlorodihydrofluorescein diacetate (DCFH-DA), propidium iodide (PI), celastrol, dimethyl sulfoxide (DMSO) and vitamin C (VC) were purchased from Sigma Aldrich (St. Louis, MO, USA). Hoechst 33342 Staining Solution for Live Cells (100×), phenylmethanesulfonyl fluoride (PMSF), anti-GAPDH antibody, HRP-labeled Goat Anti-Rabbit IgG(H+L), HRP-labeled Goat Anti-Mouse IgG(H+L), RIPA lysis buffer and primary antibody dilution buffer were purchased from Beyotime (Shanghai, China). Polyvinylidene fluoride (PVDF) membrane was purchased from Millipore (Burlington, MA, USA). Primary antibody against bcl-2, caspase-3, PARP, caspase-7 were purchased from Cell Signaling Technology (Boston, MA, USA). Anti-bax antibody was purchased from Proteintech (Chicago, IL, USA).

z-VAD-FMK was purchased from ApexBio (Houston, TX, USA). The lactate dehydrogenase (LDH) assay kit was purchased from Nanjing Jiancheng Bioengineering Institute (Nanjing, China). Cyclosporin A (CsA) was purchased from Aladdin (LA, USA). The ECL western blotting substrate was obtained from Thermo Fisher Scientific (Waltham, MA, USA).

### 2.2. Preparation of Nano-TiO_2_ Suspension

Nano-TiO_2_ was prepared into 2 mg/mL solution with PBS after ultrasonic treatment for 20 min. Then nano-TiO_2_ suspensions were diluted to work concentration and dispersed using a sonicator for 20 s again before each use.

### 2.3. UVA Irradiation

UVA light was provided by a UV lamp at 365 nm. After treated with nano-TiO_2_, the supernatant was replaced with PBS, and then irradiated with UVA at a distance of 15 cm for 1 h.

### 2.4. Cell Culture

HeLa cells were supplied by the human genome research center of Huazhong University of Science and Technology, cultured in 5% CO_2_ at 37 °C supplemented with 10% FBS and 1% penicillin/streptomycin.

### 2.5. Cell Viability

MTT assay was used to detect cell viability [23]. Cells were seeded in 96-well-plates at a density of 1 × 10^4^ cells per well and cultured for 24 h in 5% CO_2_ at 37 °C. After treated with different experiment conditions, the cells were washed with PBS three times, and then 100 μL diluent was added into each well (5 mg/mL MTT: DMEM = 1:5). After 4 h of cell culture in 5% CO_2_ at 37 °C, 150 μL DMSO was added into each well. When the crystal in the cell was fully dissolved, the microplate reader (Spectrafluor Plus, Tecan US, RTP, Durham, NC, USA) was used to detect at 492 nm.

### 2.6. Western Blot

The cells were washed with pre-cold PBS, then the RIPA lysis buffer containing 1% PMSF was added. Lysates were collected, sonicated and centrifuged at 12,000 rpm at 4 °C for 20 min. 1×loading buffer was added for boiling at 100 °C for 10 min, and then the same volume of protein was added to 12% SDS-PAGE and transferred to PVDF membrane [7]. The PVDF membrane was blocked with 5% skimmed milk for 2 h and incubated with the indicated primary antibodies overnight at 4 °C: anti-GAPDH, anti-PARP, anti-caspase3, anti-caspase7, anti-bcl2 and anti-bax were used at a 1:2000 dilution. After washing with TBST, then HRP-conjugated secondary antibodies were incubated at room temperature for 1.5 h. The reaction was developed using ECL western blotting substrate. The gray value was quantitatively estimated using the Image J software.

### 2.7. Hoechst 33342/PI Fluorescent Staining

The cells were seeded with 2 × 10^5^/well in a laser confocal culture dish. After treatment, the cells were washed twice with PBS. An amount of 5 μg/mL PI staining solution was added and incubated in dark for 30 min. After washing with PBS three times, 1× Hoechst 33342 staining solution was added for 10 min [24]. The cells were then observed under a laser confocal scanning microscope (Olympus, FV1000, Tokyo, Japan) to evaluate fluorescence intensity. The excitation wavelengths of Hoechst 33342 and PI were 405 nm and 543 nm, respectively.

### 2.8. LDH Release Detection

The LDH detection kit was used to evaluate the integrity of the cell membrane. Lactate dehydrogenase (LDH) could be released from cytosol to supernatant and measured when cell membrane damage [24]. After the cells were treated by different experiments, 25 μL supernatant was taken to be tested according to the manufacturer’s instructions.

### 2.9. Detection of Intracellular Ultrastructure

After digestion and centrifugation, the cells were fixed with 2.5% glutaraldehyde for 12 h at 4 °C and 1% osmic acid for 3 h [16]. After washing with PBS, different ethanol concentrations were dehydrated for 15 min at 30%, 50%, 70%, 90%, 95% and 100%, respectively, and 100% ethanol was dehydrated twice. The mixture of ethanol and acetone was added and soaked for 10 min. Then pure acetone was soaked twice for 15 min each time. After permeating and embedding with resin, the resins were cut into 70 nm slices, stained with uranium acetate for 30 min and lead citrate for 15 min. The images were observed and collected by transmission electron microscope (Hitachi, HT7700, Tokyo, Japan).

### 2.10. Intracellular ROS Detection

DCFH-DA probe was used to detect the intracellular ROS, which could be oxidized by ROS into fluorescent DCF after entering the cell [25]. HeLa cells were seeded into a 24-well plate at a density of 1 × 10^5^/well. After the cells had converged to 60–70%, cells were treated with different experimental conditions and washed with PBS twice. HeLa cells were added with 20 μM DCFH-DA in the dark for 30 min. After washing with PBS three times, the cells were observed under the inverted fluorescence microscope (Nikon, Japan).

### 2.11. Mitochondrial Permeability Transition Pore Detection

TMRE is a cationic cell-permeable fluorescent dye with lipophilicity. With the opening of mPTP, the fluorescence of TMRE disappeared rapidly [26]. TMRE with a working concentration of 50 nm was put into the cell culture pore and cultured with the cells in the dark for 20 min at room temperature, and then the supernatant was discarded. After 3 times of washing with PBS, the cells were observed under an inverted fluorescence microscope.

### 2.12. Statistical Analysis

All the histogram data in this paper were analyzed by GraphPad prism, and the values were expressed by mean ± SEM at least three individual experiments. The comparison between the two groups was analyzed by Student’s t-test. Values of *p* < 0.05 were considered a significant difference between the two groups.

## 3. Results

### 3.1. The Cytotoxicity and Phototoxicity of Nano-TiO_2_

**Figure 1:**
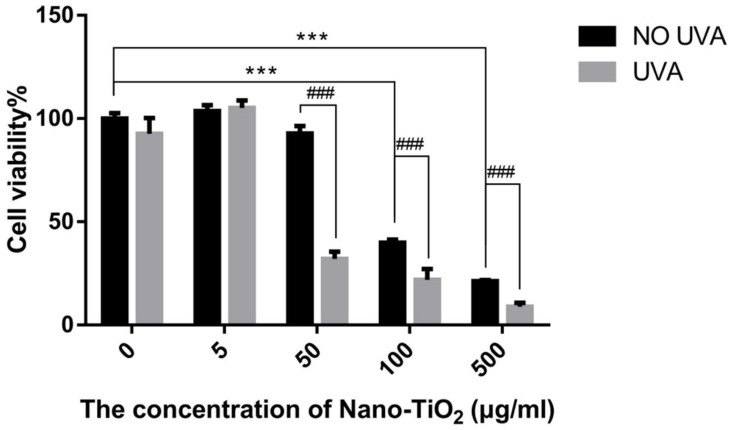
The effect of nano-TiO_2_ on HeLa cell viability with or without ultraviolet A (UVA) irradiation. HaLe cells were exposed to different concentrations (0, 5, 50, 100, 500 μg/mL) of nano-TiO_2_ for 4 h or UVA irradiation for 1 h. MTT assay was used to detect cell viability. *** *p*< 0.001, compared with 0 μg/mL group, ### *p* < 0.001, compared with the groups between nano-TiO_2_ and the same concentration of nano-TiO_2_ under UVA irradiation.

The viability of HeLa cells was tested following treatment with different concentrations of nano-TiO_2_ 4 h or UVA irradiation for 1 h (Figure 1). The results showed no significant effect on cell viability when the concentration of nano-TiO_2_ was at the range from 5 μg/mL to 50 μg/mL. However, when nano-TiO_2_ was at 100 μg/mL, the viability of HeLa cells was decreased by about 60% compared with 0 μg/mL. With the increase of concentration, the viability of HeLa cells further reduced. When the concentration of nano-TiO_2_ was increased to 500 μg/mL, the viability of HeLa cells decreased by about 80%, which indicated that the concentration of nano-TiO_2_ had a significant influence on the cell viability, and the damage of HeLa cells by nano-TiO_2_ was dose-dependent.

The phototoxicity of nano-TiO_2_ on HeLa cells was examined after UVA and nano-TiO_2_ treatment. As shown in Figure 1, there was no significant effect on cell viability when the cells were exposed to UVA alone. However, when the concentration of nano-TiO_2_ increased to 50, 100 and 500 μg/mL in the presence of UVA, the cell viability decreased by about 70%, 80% and 90% compared with 0 μg/mL, respectively. Compared with nano-TiO_2_ alone, the cell viability further decreased (*p* < 0.001), indicating that UVA significantly enhanced the cell damage of nano-TiO_2_. Thus, nano-TiO_2_ had cytotoxicity and phototoxicity effects in a concentration-dependent.

### 3.2. Detection of Cell Apoptosis by Nano-TiO_2_ under UVA Irradiation

When nano-TiO_2_ was at 50 μg/mL, there was no apparent cytotoxicity but significant phototoxicity (Figure 1). Therefore, 50 μg/mL was selected to explore the mechanism of nano-TiO_2_ phototoxicity in the following experiments.

**Figure 2:**
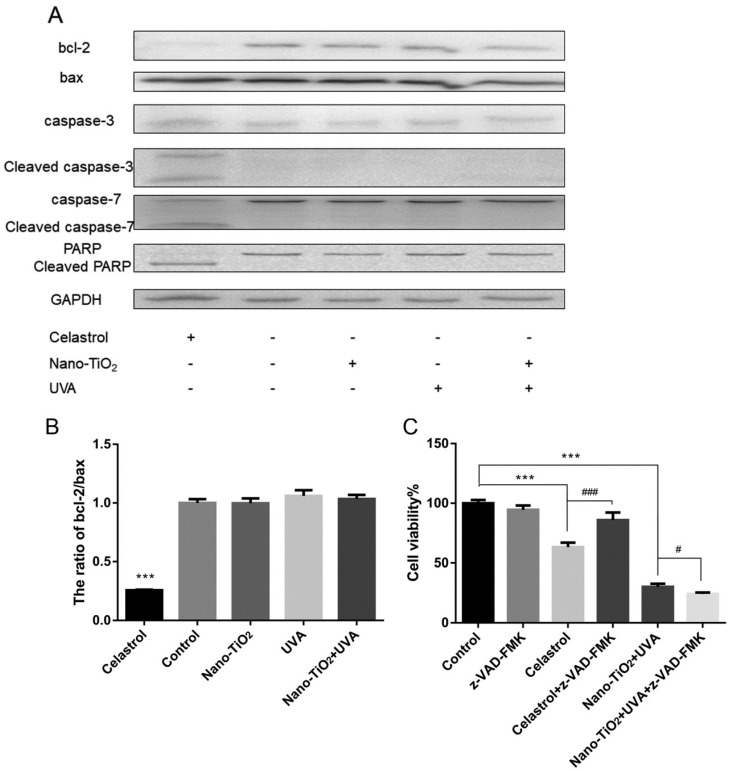
Caspase-dependent cell apoptosis was not activated in HeLa cells treated with nano-TiO_2_ and UVA irradiation. (**A**) HeLa cells were treated with 8 μM celastrol, 50 μg/mL nano-TiO_2_ or UVA irradiation, and the apoptosis-related proteins were detected by Western blot. GAPDH served as a loading control. (**B**) The data of bcl-2/bax in Western blot were statistically analyzed by Image J. *n* = 3, *** *p* < 0.001, compared with the control group. (**C**) After adding 5 μM caspase inhibitor z-VAD-FMK, the cell viability was detected by MTT assay. *n* = 5, *** *p* < 0.001, compared with the control group. ### and # means *p* < 0.001 or *p* < 0.05 respectively, compared with adding z-VAD-FMK before and after.

Celastrol is a kind natural drug for autoimmune diseases, which can induce apoptosis of various cancer cells, including HeLa cells [27,28]. Therefore, celastrol was chosen as the positive control of apoptosis in this experiment. The Western blot results revealed that the proteins of caspase-3, caspase-7 were activated, PARP was cleaved, and the expression of bcl-2/bax was down-regulated, which indicated that celastrol as a positive control induced HeLa cell apoptosis by activating the caspase-dependent signaling pathway. However, there was no significant difference in the ratio of bcl-2/bax between the control group and nano-TiO_2_ or UVA treatment alone (Figure 2A,B). Moreover, no activated caspase-3, caspase-7, and cleaved PARP were detected, which indicated that nano-TiO_2_ and UVA did not induce HeLa cells to activate the caspase-dependent apoptosis pathway.

To further verify this result, 5 μM caspase inhibitor z-VAD-FMK was added to inhibit caspase activity. It was found that the cell viability in the celastrol group was significantly increased after adding z-VAD-FMK. However, z-VAD-FMK did not change the cell viability of HeLa cells with the co-treatment of nano-TiO_2_ and UVA, but increased the death sensitivity of HeLa cells (Figure 2C). These data suggested that caspase-dependent apoptosis was not the reason for decreasing HeLa cell viability by nano-TiO_2_ under UVA irradiation.

### 3.3. Detection of Cell Necrosis by Nano-TiO_2_ under UVA Irradiation

**Figure 3:**
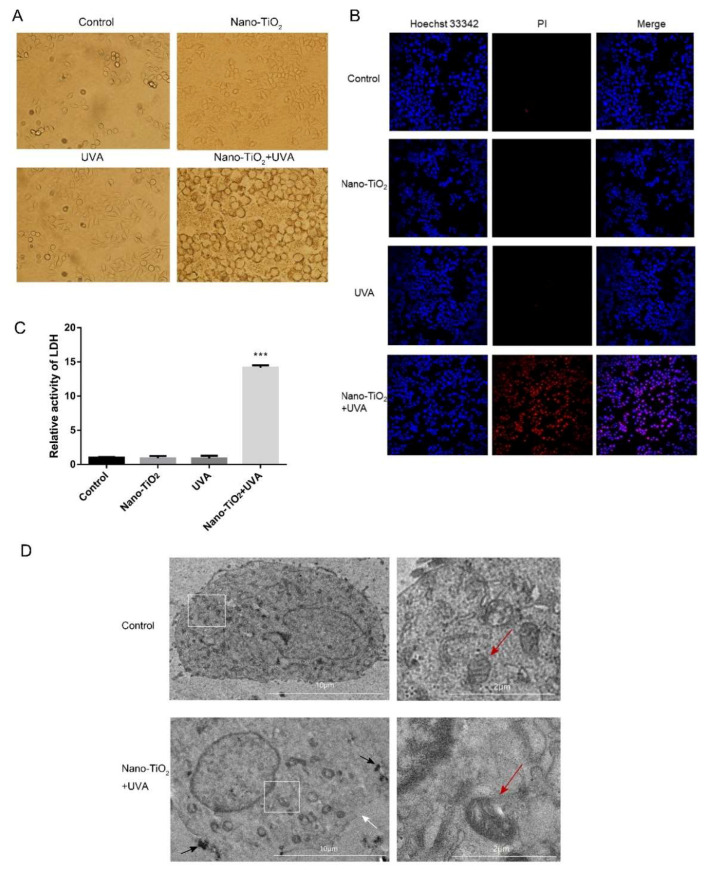
Cell necrosis induced by nano-TiO_2_ under UVA irradiation HeLa cells. (**A**) Cell morphology was observed under a light microscope. (**B**) Hoechst 33342/PI staining to detect the integrity of cell membrane. (**C**) Lactate dehydrogenase (LDH) released by cells was detected. *n* = 5, *** *p* < 0.001, compared with control group. (**D**) The ultrastructure of the cells observed by a transmission electron microscope. Black arrow represents nano-TiO_2_ in the cell; white arrow represents cell membrane rupture; red arrow represents mitochondria swelled.

Cell necrosis is a typical caspase-independent cell death mode characterized by swelling of cells or organelles, ruptured cell membranes, and transparent cytoplasm [29]. When nano-TiO_2_ was added to HeLa cells, nanoparticles could be adsorbed around the surface of HeLa cells with or without UVA. UVA irradiation or nano-TiO_2_ treatment alone did not change the morphology of cells. However, the cells showed an apparent swelling phenomenon with nano-TiO_2_ and UVA irradiation (Figure 3A).

The results of Hoechst 33342/PI staining showed no apoptotic features such as dense nuclei or fragmented DNA in HeLa cells when treated with nano-TiO_2_/UVA alone or in the co-treatment of nano-TiO_2_ and UVA irradiation, which was consistent with the results of Western blot. The red fluorescence of PI staining in nano-TiO_2_ and UVA group was significantly enhanced, which was no significant change with nano-TiO_2_ or UVA alone (Figure 3B). The LDH detection results were shown in Figure 3C. Compared with the control group, the LDH release of cells increased about 13-fold in nano-TiO_2_ and UVA group, but treated with nano-TiO_2_ or UVA alone did not change significantly. These results indicated that nano-TiO_2_ under UVA irradiation-induced HeLa cell necrosis.

To further verify the necrosis of HeLa cells induced by UVA irradiation and nano-TiO_2_, TEM was used to observe the ultrastructure of HeLa cells. As shown in Figure 3D, nano-TiO_2_ was not only adsorbed on the cell surface, but also accumulated in the cell (black arrow) because nanoparticles could enter the cells without relying on cell surface receptors [30]. Wang et al. confirmed that nano-TiO_2_ could enter human glioma cell line U87, and found that the cells began to phagocytize nano-TiO_2_ after 2 h, and formed phagocytic vesicles after 12 h [31]. The typical phenotypes of cell necrosis were observed, including HeLa cells swelled, cell membrane ruptured (white arrow), a large number of vacuoles appeared in the cytoplasm, mitochondria swelled (red arrow), and mitochondrial cristae disordered, after exposure to nano-TiO_2_ under UVA irradiation. In conclusion, HeLa cell necrosis was induced by nano-TiO_2_ under UVA irradiation.

### 3.4. The Effect of ROS on Cell Viability and Cell Necrosis

**Figure 4:**
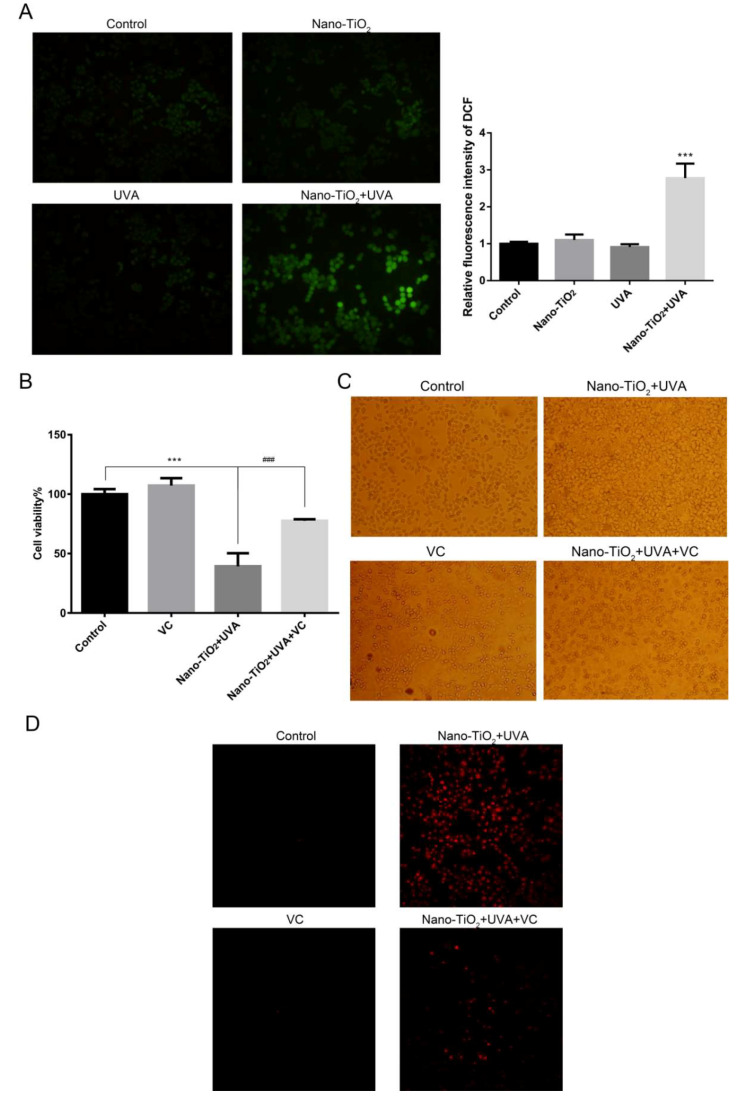
The effects of reactive oxygen species (ROS) generated by nano-TiO_2_ and UVA on cell viability and cell necrosis. (**A**)The generation of ROS was detected by 2′,7′-dichlorodihydrofluorescein diacetate (DCFH-DA) probe. The average fluorescence intensity of ROS was quantified by Image J, *n* = 3, *** *p* < 0.001, compared with control group. (**B**) MTT was used to detect the cell viability after added 0.4 mM VC, *n* = 5. *** *p* < 0.001, compared with control group; ### *p* < 0.001, compared with adding VC before and after. (**C**) The morphological changes of the cells were observed after added 0.4 mM VC. (**D**) PI fluorescence staining was used to detect the integrity of cell membrane after added 0.4 mM VC.

Although the phototoxicity mechanism of nano-TiO_2_ is still controversial, excessive ROS is regarded as a significant toxic source. DCFH-DA fluorescent probe was used to investigate the ROS level in HeLa cells. The fluorescence intensity of nano-TiO_2_ and UVA group was 2.8-fold higher than the control group. Nevertheless, there was no significant difference in the fluorescence intensity when HeLa cells were treated with nano-TiO_2_ or UVA alone (Figure 4A), which indicated that a large amount of ROS was produced by nano-TiO_2_ and UVA irradiation in HeLa cells.

Vitamin C (VC), as a common antioxidant and intracellular ROS scavenger, plays a vital role in protecting bio-membranes from oxidative damage [25]. To explore the relationship between ROS and necrosis of HeLa cells, VC was added. MTT assay showed that the cell viability in UVA and nano-TiO_2_ group increased from 39% to 77% compared with before (Figure 4B), which indicated that ROS played a critical regulatory role in the phototoxicity of nano-TiO_2_. Moreover, it was found that the cell morphology gradually reversed from swelling to normal with the additive of VC (Figure 4C). PI fluorescence staining showed that VC decreased the red fluorescence of nano-TiO_2_ and UVA group (Figure 4D), which indicated that ROS was involved in regulating cell necrosis. Reducing intracellular ROS could reduce HeLa cell necrosis effectively induced by nano-TiO_2_ under UVA irradiation.

### 3.5. The Effect of mPTP on Cell Viability in Nano-TiO_2_ Phototoxicity

**Figure 5:**
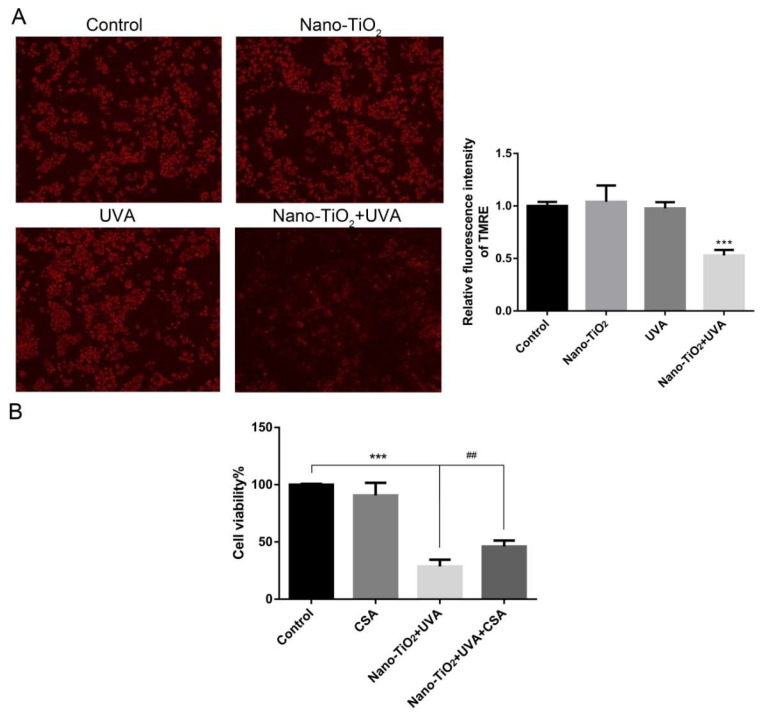
The effect of mPTP on phototoxicity of nano-TiO_2_. (**A**) HeLa cells were treated with 50 μg/mL nano-TiO_2_ or UVA irradiation, detecting mPTP opening by tetramethylrhodamine ethyl ester (TMRE) fluorescence staining. The fluorescence intensity of TMRE was statistically quantified by Image J. *n* = 3, *** *p* < 0.001, compared with the control group. (**B**) MTT assay was used to detect the cell viability after adding 0.12 mM CsA to inhibit mPTP, *n* = 5, *** *p* < 0.001, compared with the control group; ## *p* < 0.01, compared with adding CsA before and after.

To explore the role of mPTP in the process of cell necrosis induced by nano-TiO_2_ under UVA irradiation, TMRE fluorescent dye was used to detect the opening of mPTP. As shown in Figure 5A, the fluorescence intensity of HeLa cells decreased by about 50% (*p* < 0.001) by nano-TiO_2_ under UVA irradiation. However, there was no significant difference among the control group, nano-TiO_2_, or UVA treatment alone. These results indicated that the mPTP was over-opened during the damage of HeLa cells induced by nano-TiO_2_ under UVA irradiation, but the treatment of nano-TiO_2_ or UVA alone had no significant effect on mPTP.

CsA is a common specific inhibitor of mPTP opening with maintaining mitochondrial homeostasis and inhibiting cell death [26]. The results showed that CsA alone had no significant effect on the cell viability, but the cell viability was increased in nano-TiO_2_ under UVA group after added CsA (Figure 5B). These data indicated that mPTP was a vital part of the phototoxicity induced by nano-TiO_2_ under UVA irradiation in HeLa cells. Inhibition of mPTP opening can protect HeLa cells.

### 3.6. The Effect of Inhibiting ROS on mPTP

**Figure 6:**
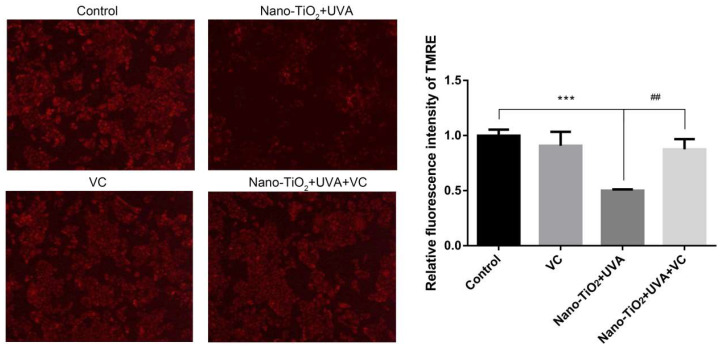
Mitochondrial permeability transition pore (mPTP) opening was relieved after inhibited ROS. HeLa cells were treated with 50 μg/mL nano-TiO_2_ and UVA irradiation, or the combination of 0.4 mM VC, nano-TiO_2_ and UVA irradiation. mPTP opening was detected by TMRE fluorescent staining. The fluorescence intensity of TMRE was statistically analyzed by Image J, *n* = 3, *** *p* < 0.001, compared with the control group; ## *p* < 0.01, compared with adding VC before and after.

The conformation of structural proteins on mPTP changes, the membrane permeability is abnormal under the stimulation of oxidative stress, which triggers downstream signal pathways and eventually causes cell death [32,33,34]. However, in the research of cell necrosis caused by acute phototoxicity of nano-TiO_2_, the relationship between ROS and mPTP remains to be studied. The red fluorescence of cells treated with nano-TiO_2_ under UVA irradiation was increased by about 50% after added VC, which indicated that the reduction of intracellular ROS could reduce the opening of mPTP, and the level of intracellular ROS was positively correlated with the opening of mPTP (Figure 6).

From the above results, the ROS level of HeLa cells increased and the mPTP was abnormally opened after the combination of nano-TiO_2_ and UVA. When the generation of ROS was inhibited, the mPTP opening was blocked and cell necrosis was decreased. It was suggested that nano-TiO_2_ under UVA irradiation regulated HeLa cell necrosis through the ROS-mPTP pathway.

## 4. Discussion

With the extensive use of nano-TiO_2_, its risk to humans is becoming more and more serious. The photocatalytic property of nano-TiO_2_ can trigger oxidative damage, destruction of cellular structures, inactivation of key proteins, and DNA break, leading to cell apoptosis or necrosis [1,33,35,36]. Therefore, it is necessary to study the phototoxicity and its mechanism of nano-TiO_2_.

HeLa cells are a standard cell line to evaluate cytotoxicity and are widely used in the nanoparticles’ toxicity, such as nano-TiO_2_, Ag/Fe_3_O_4_ nanoparticles and nano-diamond [37,38,39]. Therefore, HeLa cells were used for in vitro study of nano-TiO_2_ phototoxicity. The results showed that nano-TiO_2_ induced cell death in a concentration-dependent manner. When the concentration was increased to 100 µg/mL or 500 µg/mL, the cell viability was decreased by about 60% and 80% compared with 0 µg/mL, respectively. Simultaneously, when the concentration is ≥50 µg/mL, nano-TiO_2_ exhibited obvious phototoxicity and the cell viability decreased below 50%. With the increase of the concentration, the phototoxicity becomes higher (Figure 1).

To investigate why cell viability decreased after nano-TiO_2_ and UVA treatment, we explored the death mode of HeLa cells. Apoptotic cells are mainly accomplished by caspase activation, cell contraction, formation of apoptotic bodies, phosphatidylserine exposure outside the cell membrane, chromatin condensation, and DNA fragmentation [40,41,42]. Apoptosis related proteins levels such as Cleaved caspase-3, Cleaved PARP, Cleaved caspase-7, and bcl-2/bax, did not change significantly in nano-TiO_2_ and UVA group. The caspase inhibitor z-VAD-FMK did not increase cell viability (Figure 2). This indicated that cell death induced by nano-TiO_2_ and UVA is not due to caspase-dependent apoptosis.

Unlike apoptosis, cell necrosis is characterized by a sudden loss of cell membrane integrity, vacuoles in the cytoplasm, swelling of cells and organelles, release of cell contents, activation of immune response and inflammation of surrounding tissues, which is usually unrelated to the activation of caspase [17]. Interestingly, the swelling of cells in the nano-TiO_2_ and UVA group was observed (Figure 3A). Treatment with nano-TiO_2_ and UVA damaged the integrity of the cell membrane (Figure 3B,C). TEM results showed that nano-TiO_2_ accumulated on the cell membrane and cytoplasm under UVA irradiation, increased cell volume, cytoplasmic transparency, cell membrane rupture, mitochondria swelling, and mitochondrial cristae disorder (Figure 3D). The results indicated that nano-TiO_2_ under UVA irradiation-induced HeLa cell necrosis.

ROS is one of the main mechanisms involved in nanoparticle-induced necrosis. Under UVA irradiation, nano-TiO2 could increase the ROS level in HeLa cells (Figure 4A), consistent with the previous results [25]. Inhibition of ROS significantly increased cell viability, protected cells from swelling, and maintained the cell membrane’s integrity. It confirmed that ROS was a crucial regulatory role in the process of HeLa cell necrosis induced by nano-TiO_2_ under UVA irradiation (Figure 4B–D). Apoptosis or necrosis usually depends on the intensity or duration of the stimulation inducing death. Severe or sustained injury can cause necrosis, while light or transient stress can induce apoptosis. H_2_O_2_ produced ROS in astrocytes, phosphorylated AMPK, and increased cell necrosis [43]. Caspase inactivated by oxidative modification of cysteine residues under high oxidant conditions transformed the cells from apoptosis to necrosis [44]. ROS also made lipid peroxidation, affected energy metabolism and induced cell necrosis [45].

**Figure 7:**
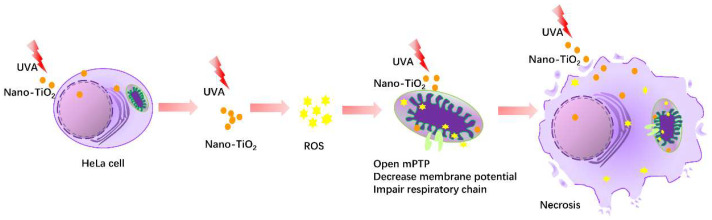
The potential mechanism of HeLa cell necrosis by nano-TiO_2_ and UVA irradiation.

The mPTP in mitochondria is involved in stabilizing mitochondrial membrane potential and balancing intracellular and extracellular ions, affecting cell necrosis and apoptosis [46]. In this study, HeLa cells displayed over-opening mPTP by nano-TiO_2_ under UVA irradiation (Figure 5A). Inhibition of mPTP increased cell viability, which confirmed the critical regulatory role of mPTP in HeLa cell death induced by nano-TiO_2_ and UVA irradiation (Figure 5B). Ca^2+^ overload or ROS stimulation caused opening mPTP, increasing mitochondrial membrane permeability, decreasing membrane potential, impairing respiratory chain function, organelle swelling and outer membrane rupture, eventually leading to cell necrosis [47]. When oxidative stress occurs, parkin located in the mitochondrial matrix can inhibit the opening of mPTP induced by H_2_O_2_ through the ubiquitination of CypD, thus inhibiting cell necrosis, reducing myocardial ischemia/reperfusion (I/R) injury and improving cardiac function [48]. Interestingly, we found the intracellular ROS inhibition alleviated the abnormal opening of mPTP induced by nano-TiO_2_ and UVA irradiation (Figure 6), indicating that ROS affects cell survival by regulating mPTP. These data suggest that nano-TiO_2_ and UVA activate the ROS-mPTP pathway to trigger HeLa cell necrosis (Figure 7).

## 5. Conclusions

In the present study, HeLa cell necrosis caused by nano-TiO_2_ and UVA and the mechanism were explored for the first time. Nano-TiO_2_ has concentration-dependent cytotoxicity and phototoxicity. The viability of nano-TiO_2_ was significantly reduced under UVA irradiation. The reason for the reduction was related to cell necrosis rather than caspase-dependent apoptosis. Moreover, HeLa cell necrosis by nano-TiO_2_ under UVA irradiation may be regulated through the ROS-mPTP pathway, which provides strong theoretical support for the safety evaluation of nano-TiO_2_ and a new idea for its toxicological mechanism. However, the HeLa cell line cannot simulate the complex physiological environment, and thus it is necessary to conduct in vivo experiments to estimate the toxicity of nano-TiO_2_ accurately.
